# Three dimensional printed models of the airway for preoperative planning of open Laryngotracheal surgery in children: Surgeon’s perception of utility

**DOI:** 10.1186/s40463-021-00524-y

**Published:** 2021-07-13

**Authors:** Oshri Wasserzug, Gadi Fishman, Narin Carmel-Neiderman, Yael Oestreicher-Kedem, Maher Saada, Solomon Dadia, Eran Golden, Philip Berman, Ophir Handzel, Ari DeRowe

**Affiliations:** 1 Pediatric Otolaryngology Unit, Dana-Dwek Children’s Hospital, Tel Aviv Sourasky Medical Center, 6 Weizman Street, 6423906 Tel Aviv, Israel; 2 Department of Otolaryngology, Head & Neck and Maxillofacial Surgery, Tel Aviv Sourasky Medical Center, Tel Aviv, Israel; 3 The Surgical 3D Printing Lab, Tel Aviv Sourasky Medical Center, Tel Aviv, Israel; 4 Sackler Faculty of Medicine, Tel Aviv University, Tel Aviv, Israel

**Keywords:** Subglottic stenosis, Likert scale, Three-dimensional printing, Direct laryngoscopy, Laryngotracheal reconstruction

## Abstract

**Background:**

Preoperative planning of open laryngotracheal surgery is important for achieving good results. This study examines the surgeon’s perception of the importance of using life size 3D printed models of the pediatric airway on surgical decision making.

**Methods:**

Life-size three-dimensional models of the upper airway were created based on CT images of children scheduled for laryngotracheal-reconstruction and cricotracheal resection with anastomosis. Five pediatric airway surgeons evaluated the three-dimensional models for determining the surgical approach, incision location and length, graft length, and need for single or double-stage surgery of seven children (median age 4.4 years, M:F ratio 4:3). They rated the importance of the three-dimensional model findings compared to the direct laryngoscopy videos and CT findings for each domain on a validated Likert scale of 1–5.

**Results:**

The mean rating for all domains was 3.6 ± 0.63 (“moderately important” to “very important”), and the median rating was 4 (“very important”). There was full agreement between raters for length of incision and length of graft. The between-rater agreement was 0.608 (“good”) for surgical approach, 0.585 (“moderate”) for incision location, and 0.429 (“moderate”) for need for single- or two-stage surgery.

**Conclusion:**

Patient-specific three-dimensional printed models of children’s upper airways were scored by pediatric airway surgeons as being moderately to very important for preoperative planning of open laryngotracheal surgery. Large–scale, objective outcome studies are warranted to establish the reliability and efficiency of these models.

**Graphical abstract:**

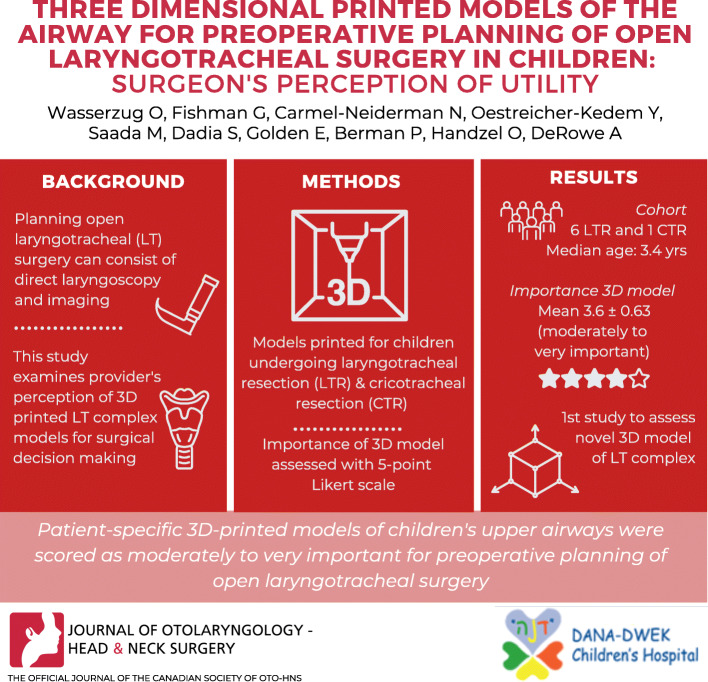

## Background

Three-dimensional (3D) printing is being employed in a variety of surgical specialties to improve patient care and is becoming standard of care in some [1]. These models enable preoperative in vitro planning [2, 3], production of tailor–made grafts and stents [4, 5], advanced training of surgical residents and improved patient education [6]. In otolaryngology, recent studies reported the use of 3D models of the larynx and trachea for laryngotracheal reconstruction (LTR) rehearsal [7], for training in endoscopic balloon dilations of subglottic stenosis [8], for creating tissue-engineered airway grafts [9, 10], and for other procedures [11–15]. To the best of our knowledge, assessment of a possible advantage of utilizing these models for preoperative planning of open laryngotracheal surgery based on a validated scoring system has not been reported. This would aid in identifying the advantages in using the technology for pre-operative planning and identify further needs for improvement.

Acquired subglottic or tracheal stenosis is not uncommon and is most commonly caused by intubation injuries [16]. Children with Cotton-Myer grades I and II stenosis are usually treated endoscopically, while LTR and cricotracheal resection with end-to-end anastomosis (CTR) are currently the preferred procedures for treatment of Cotton-Myer grades III and IV subglottic stenosis. These procedures are both challenging and require high-level surgical skills. In LTR, accurate carving of a rib cartilage graft is crucial since once carved changes are difficult to perform. Also, failure to identify the stenotic/narrowed region in the trachea or failure to expand the lumen sufficiently may result in an inadequate outcome. Dehiscence of the anastomosis after CTR can cause catastrophic sequelae.

Currently, preoperative planning of these surgeries is based on the findings on direct laryngoscopy (DL) with or without computerized tomographic (CT) scans and 3D reconstructions. When these modalities are used, single surgery and overall success rates in LTR were 75 and 87%, respectively [17]. CTR success rate is lower.

Recently, our institution has adopted the fabrication of a 3D printed model of the laryngotracheal complex before every LTR and CTR to assist with pre-operative planning and resident training. The use of the 3D model can enhance surgical planning by providing a hands-on approach with incision placement and measurements. We sought to validate our clinical impression that using the 3D model has a measurable advantage over standard pre-operative planning as perceived by the surgeon.

The objective of the current study was to evaluate whether 3D models of the laryngotracheal complex have a perceived benefit over the combined DL and CT findings for preoperative planning of open laryngotracheal surgery in children.

## Methods

### Study design, setting, and participants

IRB (Helsinki committee) approval was obtained for data collection and analysis. All children scheduled for open laryngotracheal surgery between January 2019 and February 2020 underwent pre-operative assessment including 3D models of the laryngotracheal complex, DL and low-dose CT scan.

#### 3D model

**Fig. 1:**
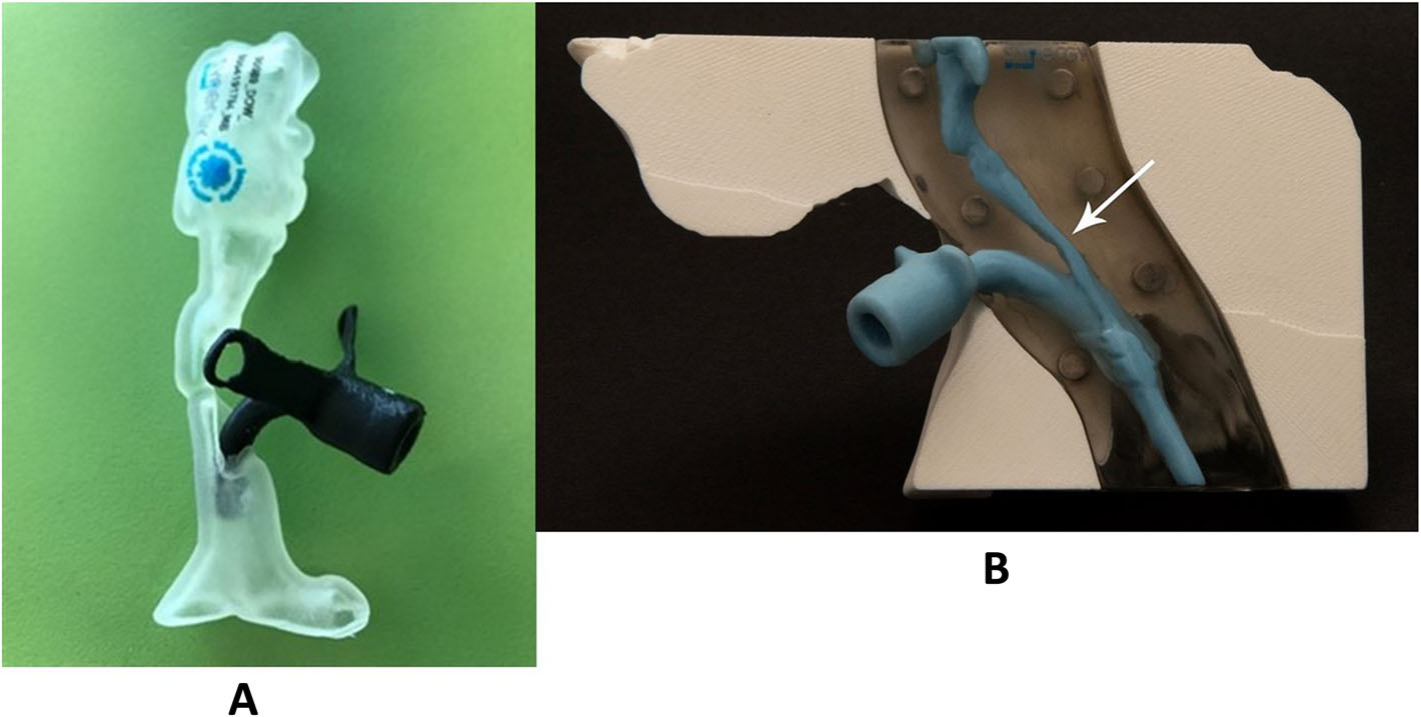
(**A**) A 3D model of complete tracheal stenosis in a child. (**B**) The same 3D model without the cannula

Life-size 3D models of the upper airway from the base of tongue to the carina were created based on CT scans of the children who were scheduled to undergo LTR and CTR. Printing of the models was based on non–contrast CT scans of the neck. The models were composed of the framework itself, the air column, the peristomal region, the tracheostomy tube, and the larynx. The software can differentiate between secretions and soft tissue, and the reproduced tracheostomy cannula is removable. A typical model is shown in Fig. 1A and B. A standard protocol was used to obtain the dimensions of the 3D-printed model from a 1-mm cuts of the CT scan. DICOM® images were securely uploaded for image processing with Mimics Medical software, version 22.0 (Materialise, Belgium), and were used to create segmentation of the airway by means of a threshold-based algorithm. Segmentation of the airway was obtained from the base of tongue to the carina. In children, tracheal rings are extremely difficult to visualize and segment, thus the models likely demonstrate the airway without the cartilage.

The preliminary segmentation results are then reviewed by the operating surgeon (or associated consultant and/or radiologist) to refine and approve. The 3D images were subsequently created and exported to 3-matic Medical software, version 14.0 (Materialise). An external shell of 2.5 mm was designed to represent the mucosa. A standard tessellation language file of the model was printed with a PolyJet J750 3D printer (Stratasys, Eden Prairie, Minnesota, USA). PolyJet employs a 3D printing technology that builds parts by jetting photopolymer droplets onto a build platform and solidifying them with ultraviolet light. The material was a proprietary material, Agilus 30™ (Stratsys, Rehovot, Israel). The process resulted in the formation of soft and flexible 3D models that depicted the area of stenosis and had a similar tactile feel as cartilage and scar tissue.

In order to evaluate the utility of the pre-operative 3D model, four fellowship-trained pediatric otolaryngologists (O.W., G.F., M.S. A.D.) and one fellowship trained laryngologist (Y.O-K.) who regularly perform open airway surgery in our institution were asked to evaluate whether there was any advantage in utilizing 3D models compared to the combined use of DL videos and CT scans. None of the surgeon’s had prior experience with the use of pre-operative 3D models. For each case, the surgeons first viewed the DL video and the CT images, after which they were given the 3D model of the same case. This was done individually, and the surgeons were not aware of the scoring documented by the others. The evaluation was performed regarding the addition of the 3D model with a Likert scale for importance [7, 18, 19], in which a score of 1 = “not at all important”, 2 = “slightly important”, 3 = “moderately important”, 4 = “very important” and 5 = “extremely important”. The additive value of the models was rated across five domains: the type of surgery required (question 1), the location of the incision (question 2), the length of the incision (question 3), the length of the graft (question 4), and the need for either single- or double-stage surgery (question 5).

### Statistical analyses

Statistical analyses were performed with NCSS 2020 (version 20.0.1 Statistical Software. NCSS, LLC. Kaysville, Utah, USA, 2020) and R (version 3.6.1, R Foundation for Statistical Computing, Vienna, Austria, 2019). The mean and median of each question was assessed. Agreement between surgeons was observed by means of Fleiss Kappa statistics. Agreement was observed twice, first by using cutoff values of ≥3 and then by using cutoffs of ≥4. Reference values for Kappa were compiled according to Landis and Koch [20]. Values between 0.4 and 0.6 represent “moderate agreement” and values between 0.6 and 0.8 represent “good agreement”. A histogram described the distribution of the answers in all evaluations.

## Results

Seven children underwent LTP (*n* = 6) and CTR (*n* = 1) with CT scans served to create the 3D models during the study period, the median age of the seven children was 3.4 years. The M:F ratio was 4:3. Five children had subglottic stenosis grade III, one had subglottic stenosis grade II, and one had grade IV complete stenosis. Table 1 summarizes the results of the Likert scale scores. The mean rating for all domains was 3.6 ± 0.63, which means that the raters found the additive value of the 3D models to be “moderately important” to “very important”, with a trend towards “very important”. The median for all domains, all models, and all raters was 4 (“very important”).

**Table 1:** Likert Scale: Mean, Standard Deviation (SD), and Median Scores for Each Domain According to the Five Domains for All Seven Models And for All Five Raters

Domain	Mean score (SD)	Median
1. Type of surgery required (LTP or CTR)	3.29 ± 0.95	3
2. Location of the incision	3.58 ± 0.58	4
3. Length of the incision	3.2 ± 0.77	3
4. Length of the graft	3.91 ± 0.41	4
5. Need for a 1- or 2-stage procedure	3.9 ± 0.55	4
Sum of all domains	3.6 ± 0.63	4

*LTP* laryngotracheoplasty, *CTR* cricotracheal resection with anastomosis

The mean value was 3.29 (“moderately important” to “very important”) for the type of surgery (question 1), 3.58 (“moderately to very important”) for the location of the incision, 3.2 (“moderately to very important”) for the length of the incision, 3.9 for the length of the graft, and 3.91 for the need for single or double-stage surgery (“moderately important”) (Table 1). The median was 3 (“moderately important”) for questions 1 and 3, and 4 (“very important”) for questions 2, 4 and 5.

**Fig. 2:**
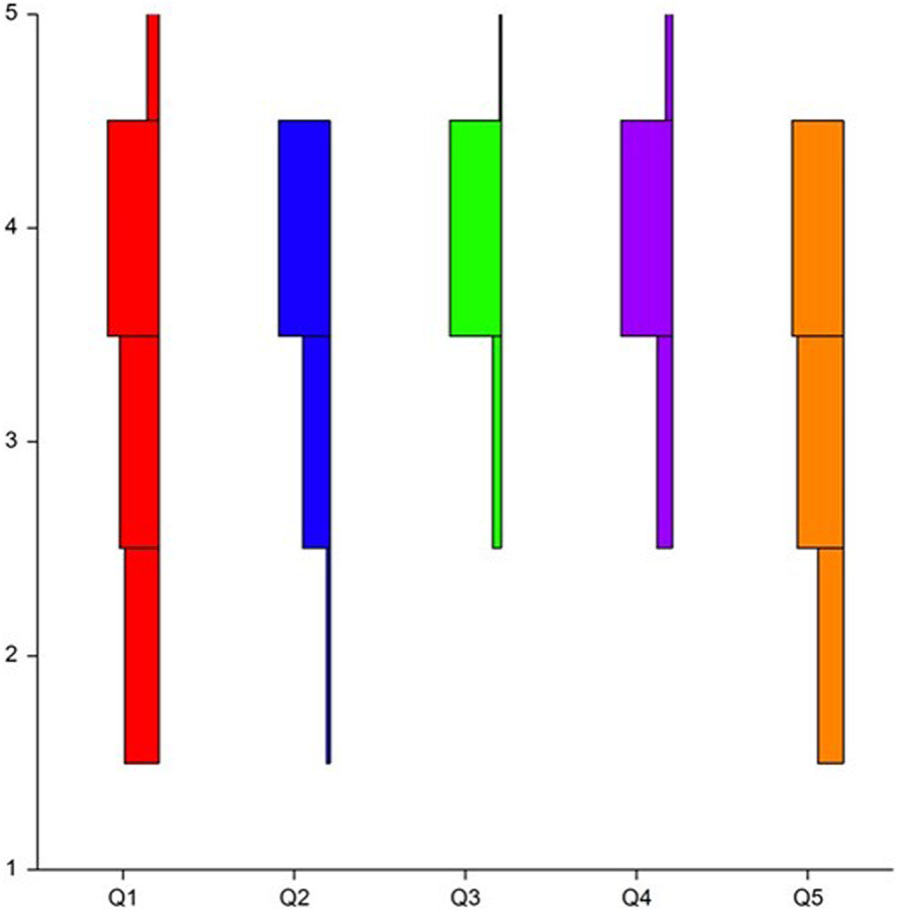
A histogram showing the distribution of the Likert scale scores for importance by all five raters for all five questions (Q1–5)

The histogram in Fig. 2 shows the distribution of the Likert scale scores for importance by all raters for all seven models and all five questions. Agreement between raters was tallied twice, first using a cutoff of ≥3 and second using a cutoff of ≥4, according to Landis and Koch Reference values for Fleiss Kappa statistics [20]. There was total agreement of the scores between the raters for questions 3 and 4. The agreement between raters was 0.608 (good) for question 1, 0.585 (moderate) for question 2, and 0.429 (moderate) for question 5.

## Discussion

The technology of 3D printing is promising in the field of pediatric otolaryngology. In the present study we show that patient-specific 3D printed models of the upper airway were scored by pediatric airway surgeons as being moderately to very important for preoperative planning of open laryngotracheal surgery. This is the first study to investigate whether there is any advantage for the use of novel 3D models of the laryngotracheal complex as an addition to DL findings and CT images.

The use of medical 3D printing is gaining popularity in many surgical specialties in parallel with the exponential growth of the technology that enables it. Patient- specific image data are used to create 3D-printed models that can be used to either simulate and plan the surgery or to simulate in-vivo grafts or splints. In addition, parents can hold the 3D model in their hands and visualize both the pathology and the surgical method that will be used to correct it, thus obtaining precise information on what to expect and what will be done in the operating room.

In the field of otolaryngology, 3D printing has been reported in many subspecialties. In otology, 3D-printed models of the mastoid and middle ear are used for preoperative planning of complicated cases of recurrent cholesteatoma [21, 22], to produce hearing aid molds [5] and to create temporal bone models for trainee education. In sinus and skull base surgery, 3D models are used to model endoscopic sinus surgery [23], and for preoperative petro-clival tumor resection planning [24]. Rose et al. [13] reported their results with the employment of a head-worn augmented reality system for intraoperative localization of pathology and normal anatomy during head and neck surgery. The average error in measurement of accuracy was only 2.47 ± 0.46 mm, making its application feasible for improvement of surgical efficiency and patient safety in the operating room. In plastic surgery for congenital craniomaxillofacial anomalies, one example of the application of 3D-printed molds is to design and sculpt a new ear based on the normal contralateral ear [25]. In a recent review [26], 3D printed models were evaluated for endoscopic surgical planning of tracheal stenosis in adults. The reported advantages included a decrease in operating time and increase in the accuracy of choosing stent diameters and placement. We present the surgeons perspective using 3D airway models in regard to the planning and decision making process in open laryngeal-tracheal surgery in children.

In pediatric otolaryngology, Zopf et al. [27] were the first to report the use of 3D printing to practice the placement of a 3D-printed airway splint in an infant with tracheobronchomalacia. Morrison et al [28] described their experience with implanting 3D-printed external airway splints in three infants with tracheobronchomalacia. Balakrishan et al. [29] reported the impact of 3D printing on surgical planning in a case series of five children who underwent open airway surgery. Richard et al. [3] described the usefulness of preoperatively practicing resection and anastomosis of 3D models of two children with tracheal stenosis.

We conducted a study aimed at quantifying a potential advantage of 3D-printed models in addition to DL and CT findings for preoperative planning of open laryngotracheal surgery in children. We decided to use the Likert scale as it is a well-validated tool, but also because we found its “Importance” variant to be highly suitable for complex airway cases. The mean score for all five domains was 3.6 ± 0.63, meaning that the raters found the advantage of the 3D models to range from “moderately important” to “very important”, with a trend towards “very important”. The median score was 4, which corresponds with “very important”. The histogram in Fig. 1 demonstrates that the bulk of the scoring was located around the score of 4 for all five domains. It could therefore be concluded that the raters found the models to be “very important” for the most part.

The highest scores were for “length of the graft” and for “need for a one- or two-stage procedure” (3.9 for both). An in-vitro measurement of the length of the stenotic region and accordingly the length of the required graft is probably more accurate than an in-vivo measurement because of the ability to cut the 3D model of the trachea completely and perform direct, precise measurements.

We consider the dilemma of whether to perform a one- or two– stage procedure to be the most challenging decision in open laryngotracheal surgery, and any assistance to that decision-making process is highly warranted. Indeed, that element may represent the greatest advantage of the 3D models in this setting, i.e., the ability to see the airway as a whole and not “slice by slice”. In addition, the ability to perform precise measurement of the distance between the vocal cords and the stenotic region and between the stenotic region and the stoma can also guide the decision of whether a one- or two– stage procedure is optimal surgery for a specific child. Although measurements of the airway can be attained endoscopically and with imaging, surgeons are trained to incorporate tactile feedback during the surgical procedure. The 3D model appears to enable pre-operative simulation thus a better understanding of these dimensions on surgical decision making.

The domain “length of the incision” had the lowest score (3.2), which correlates with “moderately important”. Indeed, the incision in the trachea is probably best determined by the findings during DL, and once the trachea has been opened, the surgeon can directly identify the site of the stenosis and make the length of the incision accordingly.

The agreement between the five raters was “complete” for domains 3 and 4, “good” for domain 1, and “moderate” for domains 2 and 5. We intend to further improve the models, based on the comments of the surgeons who gave low scores in these domains.

New technology is always interesting and appealing, but it is important to measure its true value, and whether it is essential or just “nice to have”. Both the software and the hardware of the 3D printers are improving with time. They are now more precise than they were one year ago, and the texture of the materials that are used to fabricate the models are becoming more and more realistic, almost giving the feel of real human tissue. In our institution, the cost of the 3D model is US$95 for each patient. In-house printing costs are approximately 5 USD (material only), The surgeon’s input requires about 15 min therefore can be neglected, engineer’s labor is about 2 h (including segmentation, modeling, and post processing). Outsourcing of this process will increase the costs to about 300 to 450 USD per model. Hence, we believe that these models will become more useful and common in practice. Studies that measure the benefit of 3D models for preoperative planning of open laryngotracheal surgery in children in terms of decannulation rates and postoperative complications rates are essential, however, due to the variability in the pathologies and the presence of neurological and pulmonary comorbidities in many of these children, objective outcome measures have yet to be defined.

The need for a CT scan to create the models could pose a problem, although cervical CT is suggested in cases of grade IV subglottic stenosis in order to assess the length of the segment to be resected. Our practice is to perform CT scans in children with Cotton-Myer grade IV complete obstruction or Cotton grade III with obstruction approaching 80%, as well as in children with two levels of stenosis when the upper stenosis is too severe to enable visualization of the distal segment during DL. 3D model of the upper airway based on magnetic resonance imaging (MRI) studies can potentially replace CT scanning. Indeed, we have recently created a 3D model of the upper airway based on MRI studies, and intend to continue with the attempt to create the future 3D models based on MRI imaging.

In 2018, the Radiological Society of North America published guidelines for medical 3D printing in order to provide recommendations for consistent and safe fabrication of 3D models based on imaging studies [1]. The process of creating a 3D-printed model based on imaging studies includes segmentation, which is usually done by the surgeon together with the 3D printing engineer. The surgeon delineates the borders of the lesion for the engineer and the model is produced accordingly. As such, a high level of accuracy of the segmentation of the lesion, whether it is a tumor, stenosis, or cholesteatoma, for example, is vital. The production of an inaccurate model can lead to crucial mistakes in surgical planning. Tested and validated manufacturing practices should, therefore, be implemented throughout the fabrication process [30, 31].

There are several limitations in our study that bear mention. First, the reliability of the 3D models is dependent upon the operator’s level of accuracy in creating the segmentation based on the patient’s CT images, making it is inherently subjective and at risk of varying from patient to patient. Second, 3D printing technology is expanding dramatically and materials that are currently being used in creating these models may soon be replaced by new materials that will be more realistic in their texture and of greater fidelity to the imaging study upon which they were based. Third, the surgical outcome measures of our patients were not recorded, and we do not know whether these models can affect the rate of de-cannulation, which is the main goal of open laryngotracheal surgery. Fourth, the number of children included in the study, seven, may appear inadequate. However, each case was assessed by 5 operators thus multiplying the data points that were compared using valid statistical methods. Further limitations are rooted in the study design: Using subjective measures of utility involves bias. Inclusion of measurable outcome measures would add validity to the study but would require a large cohort to have any statistical relevance. The purpose of the study was to see if the surgeon experienced in airway reconstruction found any advantage in pre-operative planning with 3D models of the airway**.**

## Conclusions

The technology of 3D printing is well established and holds promise for improving outcomes in complicated airway surgery. It enables the creation of complex patient-specific models of complex structures in the field of pediatric otolaryngology, but its importance to the surgeon as a tool for preoperative planning has not yet been assessed. In our study, patient-specific 3D printed models of the upper airway were found by pediatric airway surgeons to be from moderately important to very important for preoperative planning of open laryngotracheal surgery. Further large–scale, objective outcome studies are required to establish the reliability and efficiency of these models. With the rapid evolution of this technology, these models are expected to become even more accurate than they are today, their texture will become more realistic, and their application may very well become common practice.

## Data Availability

All patient data is on the institution’s electronic medical records.

## Abbreviations

3D: Three dimensional
CT: Computed Tomography
MRI: Magnetic Resonance Imaging
DL: Direct Laryngoscopy
LTR: Laryngotracheal reconstruction
CTR: Cricotracheal resection and anastomosis

## Declarations

IRB (Helsinki committee) approval was obtained for retrospective data collection and analysis.
